# A topographical atlas of α-synuclein dosage and cell type-specific expression in adult mouse brain and peripheral organs

**DOI:** 10.1038/s41531-024-00672-8

**Published:** 2024-03-19

**Authors:** Haley M. Geertsma, Zoe A. Fisk, Lillian Sauline, Alice Prigent, Kevin Kurgat, Steve M. Callaghan, aSCENT-PD Consortium, Benjamin R. Arenkiel, Brit Mollenhauer, Michael G. Schlossmacher, Christine Stadelmann, Julianna J. Tomlinson, Michael X. Henderson, Maxime W. C. Rousseaux

**Affiliations:** 1 University of Ottawa Brain and Mind Research Institute, Ottawa, ON K1H8M5 Canada; 2 Department of Cellular and Molecular Medicine, University of Ottawa, Ottawa, ON K1H8M5 Canada; 3 Aligning Science Across Parkinson’s (ASAP) Collaborative Research Network, Chevy Chase, MD USA; 4 Department of Neurodegenerative Science, Van Andel Institute, Grand Rapids, MI USA; 5 Department of Molecular and Human Genetics, Baylor College of Medicine, Houston, TX 77030 USA; 6 Paracelsus-Elena-Klinik, Kassel, Germany; 7 Program in Neuroscience, Ottawa Hospital Research Institute, University of Ottawa Brain and Mind Research Institute, Ottawa, ON K1H8M5 Canada; 8 Institute of Neuropathology, University Medical Center Göttingen, Göttingen, Germany

**Keywords:** Parkinson's disease, Neurodegeneration

## Abstract

Parkinson’s disease (PD) is the second most common neurodegenerative disease worldwide and presents pathologically with Lewy pathology and dopaminergic neurodegeneration. Lewy pathology contains aggregated α-synuclein (αSyn), a protein encoded by the *SNCA* gene which is also mutated or duplicated in a subset of familial PD cases. Due to its predominant presynaptic localization, immunostaining for the protein results in a diffuse reactivity pattern, providing little insight into the types of cells expressing αSyn. As a result, insight into αSyn expression-driven cellular vulnerability has been difficult to ascertain. Using a combination of knock-in mice that target αSyn to the nucleus (*Snca*^*NLS*^) and in situ hybridization of *Snca* in wild-type mice, we systematically mapped the topography and cell types expressing αSyn in the mouse brain, spinal cord, retina, and gut. We find a high degree of correlation between αSyn protein and RNA levels and further identify cell types with low and high αSyn content. We also find high αSyn expression in neurons, particularly those involved in PD, and to a lower extent in non-neuronal cell types, notably those of oligodendrocyte lineage, which are relevant to multiple system atrophy pathogenesis. Surprisingly, we also found that αSyn is relatively absent from select neuron types, e.g., ChAT-positive motor neurons, whereas enteric neurons universally express some degree of αSyn. Together, this integrated atlas provides insight into the cellular topography of αSyn, and provides a quantitative map to test hypotheses about the role of αSyn in network vulnerability, and thus serves investigations into PD pathogenesis and other α-synucleinopathies.

## Introduction

Parkinson’s disease (PD) and related dementias exist along a clinical and pathological continuum, sharing a common protein pathology. α-synuclein (αSyn) undergoes aggregation and is thought to contribute to disease pathogenesis in several neurodegenerative diseases (i.e. PD, Lewy body dementia [LBD], multiple system atrophy [MSA]), collectively termed α-synucleinopathies^1,2^. αSyn accumulates in proteinaceous structures called Lewy bodies and Lewy neurites (collectively: Lewy pathology) except for MSA where it accumulates in glial cytoplasmic inclusions ([GCIs])^3,4^. Mutations and copy number gains in the gene encoding αSyn, *SNCA*, can also cause PD^5,6^. Thus, the convergence of pathology and genetics place αSyn at the center of pathogenesis for these disorders. Despite its linkage to PD over 25 years ago, less is known about the physiological role of αSyn and how it transitions from a native, functional form to a pathogenic state^7^. This transition and its cell-selective formation of pathology are important not only to neuronal α-synucleinopathies, such as PD and LBD, but also to the oligodendrogliopathy, MSA^8^. Normally, αSyn is found at the presynaptic membrane, and to lesser extents in the nucleus, mitochondria, and cytosol^9–13^. In disease conditions, αSyn mis-localizes from the membrane to cytosolic compartment where it adopts beta-sheet fibrillar conformations^14–16^. How initial misfolding events are linked to the ultimate formation of Lewy pathology remains unclear.

Increasing evidence suggests that expression levels of αSyn, as well as posttranslational modifications, conformational changes, and cellular milieu all play a role in modulating its aggregation^5,6,17,18^. Biochemical and biophysical data have begun to establish key modifications and conformations that lead to αSyn accumulation and aggregation^19–21^. Moreover, increasing data suggest that misfolded αSyn may act in a prionoid manner, spreading from one cell to another, initiating aggregate formation^22,23^. This process requires both a template and substrate and therefore is dependent on αSyn expression in the target cell^24–26^. Yet, topographical measurement of αSyn expression has been relatively understudied to date. This may be in part due to the limited tools available to glean meaningful insight at sufficient resolution. Since αSyn is largely localized to synapses, immunodetection in healthy individuals or model organisms results in presynaptic staining, making it difficult to attribute expression to any particular cell. RNA detection methods such as fluorescence in situ hybridization have offered insight into this distribution, but there is no quantitative *Snca* map using this technique. Furthermore, it is important to recognize that cellular RNA and protein levels may not always correlate, thereby emphasizing the importance of a comprehensive brain-wide comparative analysis encompassing both proteomic and transcriptomic dimensions^27^.

We recently developed a *Snca*^*NLS*^ mouse allele to study the consequence of αSyn nuclear mislocalization^28^. These mice have a nuclear localization sequence (NLS) and Flag epitope tag knocked into the C-terminus of the endogenous *Snca* gene that codes for αSyn. While characterizing these mice, we serendipitously observed that targeting the protein to the nucleus provides unprecedented detail on the cellular and anatomical topography of this predominantly presynaptic protein. We took advantage of this cell body localization to map the regional and cellular localization of αSyn throughout the brain on the protein level and compared this to *Snca* RNA. Because the *Snca*^*NLS*^ mice show age-dependent phenotypes, we exclusively used young adult mice to capture αSyn localization without confounding neurodegenerative effects. We present a systematic study of its topography throughout the brain, spinal cord, retina, and enteric nervous system. We find that αSyn is broadly expressed in neurons of both the central and enteric nervous system (CNS and ENS, respectively) with specific region and neuron type enrichment. In addition, we find that αSyn is expressed in a subset of Olig2-positive cells, likely representing oligodendrocyte precursor cells. To our surprise, some cell clusters including ChAT-positive cells of the lateral reticular nucleus (LRN) and spinal cord motor neurons do not express αSyn.

## Results

### Whole-brain mapping reveals distinct domains of anatomical αSyn expression and distribution

To study the global topography of αSyn across the mouse brain, we used four parallel approaches, all of which use the *Snca*^*NLS*^ knock-in mice unless otherwise noted: (1) *Snca* RNA mapping in wild-type mice, (2) regional protein mapping with immunofluorescence and immunohistochemical staining, (3) whole brain clearing and immunolabeling, and (4) cell type marker co-localization.

**Fig. 1:**
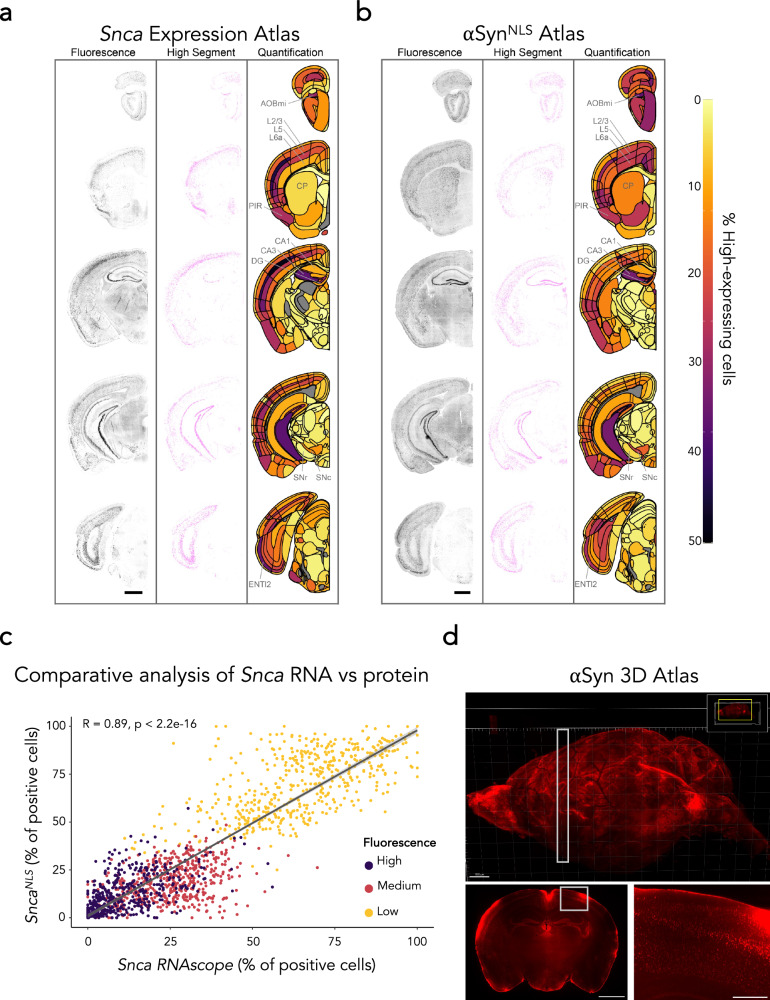
A brain-wide atlas of αSyn topography. RNAScope performed on a wild-type mouse to measure *Snca* expression (**a**) and immunofluorescent staining performed on a mouse brain measuring αSyn protein density (**b**) throughout the brain. Staining (left), segmentation (middle, high threshold), and heatmap (right) show brain region-specific expression patterns of *Snca* or αSyn distribution, respectively. Select regions with a large percentage of high-expressing cells (AOBmi accessory olfactory bulb, mitral layer, PIR piriform area, BLAa basolateral amygdalar nucleus, anterior part, CA3 field CA3 of hippocampus, DG dentate gyrus, SNc substantia nigra, compact part, ENTl2 entorhinal area, lateral part, layer 2) or lower percentage (CP caudoputamen, CA1: field CA1 of hippocampus, SNr substantia nigra, reticular part) are labeled. Cortical layers are also highlighted since these show clear differences in the percentage of high-expressing cells (L2/3: layer 2/3, L5: layer 5, L6a: layer 6a). Scale bars: 1000 μm. **c** A comparative analysis of (**a**) and (**b**) indicating correlation of topography between *Snca* RNAScope in wildtype mice and αSyn immunofluorescence in *Snca*^*NLS*^ mice for different intensity cut-offs. Dots represent class prevalence from individual regions. The solid line represents the line of best fit, and the shaded ribbons represent the 95% prediction intervals. The R and *p* values for the Pearson correlation between protein and gene expression are noted on the plots. **d** Representative images of whole brain Flag epitope staining and imaging provide an encompassing view of αSyn topography. White boxes indicate insets. Scale bars: 1000 μm (d top, d bottom left), 250 μm (d bottom right).

We previously found that the *Snca*^*NLS*^ mice have a 50% reduction in *Snca* RNA and protein, therefore we first aimed to determine if the topography of αSyn in the *Snca*^*NLS*^ mice is similar to *Snca* mRNA expression in wildtype mice. To determine the cellular expression of αSyn, we first stained for *Snca* mRNA in wildtype mouse brain sections (Fig. 1a). These sections were then put through a cell classifier in QuPath to classify cells as either high (greater than one standard deviation above the mean), medium (one standard deviation above or below the mean), or low (one standard deviation or more below the mean) *Snca* expression. (Supplementary Fig. 1a).

Second, we performed serial sectioning and diaminobenzidine (DAB) staining for αSyn at defined coordinates, then quantified the relative density of αSyn+ cells throughout the brain. The DAB-stained tissue from both coronal (Supplementary Fig. 2a) and sagittal (Supplementary Fig. 2b) sections were analyzed using ilastik, an image classification and segmentation tool that uses machine learning to delineate αSyn+ cells^29^. Next, the αSyn+ cell density was averaged against the hematoxylin+ cell density to obtain the relative αSyn+ cell density per brain region (Supplementary Fig. 2c).

Third, to better understand the regional density of αSyn+ cells based on their expression levels, we generated a coronal atlas from IF-stained brain tissue at defined coordinates (Supplementary Fig. 1b). This tissue was analyzed using an adapted version of the QUINT workflow^30^, which uses QuPath, QuickNII^31^, VisuAlign^32^, and Nutil^33^ to quantify cell classes in brains registered to the Allen Brain Atlas CCFv3 (Fig. 1b). Since the *Snca*^*NLS*^ model relies on functional importin proteins to harness the NLS tag and translocate αSyn into the nucleus for detection, we compared this αSyn^NLS^ atlas to the *Snca* RNA atlas. In all methods tested, we observe concordant results suggesting that *Snca* RNA broadly correlates with αSyn protein (Fig. 1c).

Fourth, we performed whole-brain staining for αSyn, using the Flag epitope present in *Snca*^*NLS*^ mice, coupled with light sheet microscopy to generate 3D renderings of αSyn topography throughout an intact brain (Fig. 1d). Using this, we further explored the αSyn+ density and intensity from an intact brain, which showed similar expression patterns to our other methods (Supplementary Fig. 2d). In all methods, we found high αSyn expression throughout the cortex, especially cortical layers 4 and 5, with lower expression generally in layers 2/3 and 6. We also found high αSyn density and intensity in the hippocampus, amygdala, and olfactory bulb, including the mitral, granule, and glomerular cell layers. αSyn expression was notably low in most thalamic and mesencephalic nuclei, except for the substantia nigra pars compacta (SNc).

### Neurons express variable αSyn levels

**Fig. 2:**
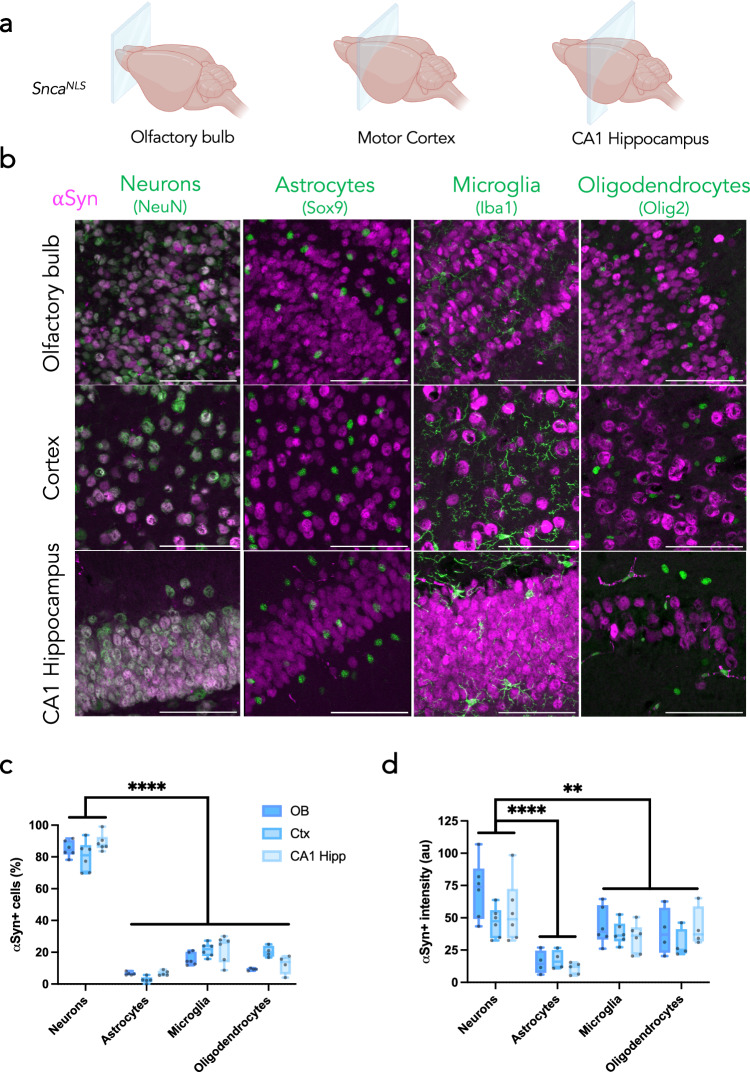
αSyn is predominantly expressed in neurons. **a** Coronal planes chosen to assess different brain regions in *Snca*^*NLS*^ mice. **b** Merged micrographs from the olfactory bulb (upper), motor cortex (middle), and CA1 hippocampus (bottom) staining for neurons (far left), astrocytes (middle left), microglia (middle right), and oligodendrocytes (right). Scale bars: 75 μm. From these, αSyn density (**c**) and intensity (**d**) were quantified. Plotted as box plots with min to max bars. Two-way ANOVA with Tukey’s post hoc, **, **** denote *p* < 0.01 and *p* < 0.0001, respectfully.

Pathological αSyn accumulates in multiple cell types, including monoaminergic neurons, glutamatergic neurons, and oligodendrocytes^34–36^. One hypothesized reason for this is that susceptible cells harbor more αSyn, thus rendering them more vulnerable to the aggregation process. The most common α-synucleinopathy, PD, is characterized by aggregates within neurons; however, a rarer α-synucleinopathy, MSA, is characterized by oligodendroglial cytoplasmic inclusions^2,4^. We sought to characterize αSyn-expressing cell types in brain regions with high αSyn+ cell density, namely the olfactory bulb, motor cortex, and hippocampus (Fig. 2a). We co-stained *Snca*^*NLS*^ mice for αSyn with cell-type markers for neurons (NeuN), astrocytes (Sox9), microglia (Iba1), and oligodendrocytes (Olig2; Fig. 2b). Congruent with previous studies, αSyn is a primarily neuronal protein, where ~ 80% of neurons in these brain regions consistently co-stain for αSyn, whereas only 10–20% of astrocytes, microglia, and oligodendrocytes express αSyn to a modest degree (Fig. 2c). Neurons also express significantly more αSyn than all other cell types tested. Among the other cell types, microglia and oligodendrocytes have similar intensities of αSyn between one another, both generally higher than that of astrocytes (Fig. 2d).

### Monoaminergic cells throughout the brain broadly and consistently express αSyn whereas cholinergic neurons show variability

**Fig. 3:**
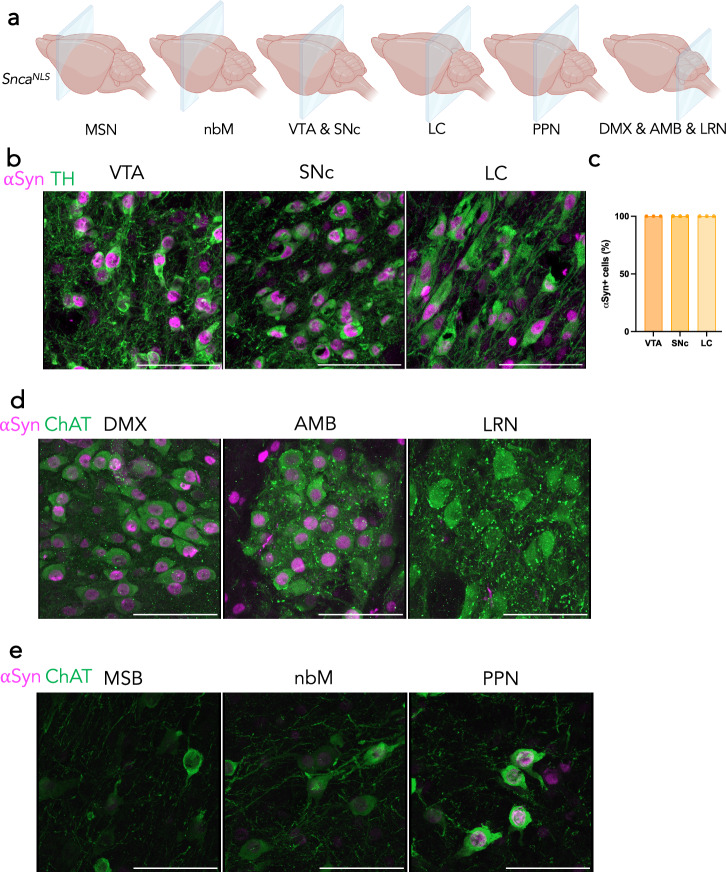
αSyn is highly expressed in catecholaminergic and cholinergic cell types that are vulnerable in PD. **a** Coronal planes chosen to assess different brain regions in *Snca*^*NLS*^ mice. **b** TH+ cells of the ventral tegmental area (VTA, left), substantia nigra *pars compacta* (SNc, middle), and locus coeruleus (LC, right) with (**c**) quantification of αSyn+ cell density. **d** ChAT+ cells of the dorsal motor nucleus of the vagus nerve (DMX; left), nucleus ambiguous (AMB; middle), and lateral reticular nucleus (LRN; right). **e** ChAT+ cells of the medial septal nucleus (MSN; left), nucleus basalis (nbM; middle), and pedunculopontine nucleus (PPN; right). Scale bars: 75 μm.

Since PD is characterized by selective dopaminergic neurodegeneration—and more broadly, monoaminergic susceptibility^37^– we explored the density and intensity of αSyn protein in monoaminergic (TH+ ) cells of the ventral tegmental area (VTA), SNc, and locus coeruleus (LC; Fig. 3a, b). In PD, the SNc and LC have significant dopaminergic neurodegeneration, but the VTA is reported to have relatively less dopaminergic degeneration^37^. Interestingly, we found that 100% of TH+ neurons in all three brain regions co-stain for αSyn (Fig. 3b, c). Another brain region that is of importance in αSyn spreading, and PD degeneration is the dorsal motor nucleus of the vagus nerve (DMX). When staining for cholinergic neurons, we also found that 100% of ChAT+ cells of the DMX show αSyn+ staining (Fig. 3d). The DMX-adjacent nucleus ambiguous (AMB), which is not known to accumulate αSyn pathology in PD^37^, was also positive for αSyn (Fig. 3d). Intriguingly, we identified other ChAT+ cells, including the lateral reticular nucleus (LRN), that were devoid of αSyn+ staining (Fig. 3d), suggesting that αSyn expression is not part of the essential ChAT+ neuron gene expression pattern. Despite this, the LRN has been shown to harbor significant αSyn pathology in disease, which may support the prion-like spreading hypothesis of αSyn to this particular brain region in disease^34,38^. We explored other ChAT+ brain regions that are known to harbor αSyn pathology and degenerate in PD, including the medial septal nucleus (MSN)^39^, nucleus basalis of Meynert (nbM)^40^, and the pedunculopontine nucleus (PPN)^41^. These regions had variable levels of αSyn, however most of the cells were αSyn+ , with the PPN having the brightest αSyn+ cell intensity of these three regions (Fig. 3e).

### Spinal cord neurons exhibit heterogeneous αSyn expression patterns

**Fig. 4:**
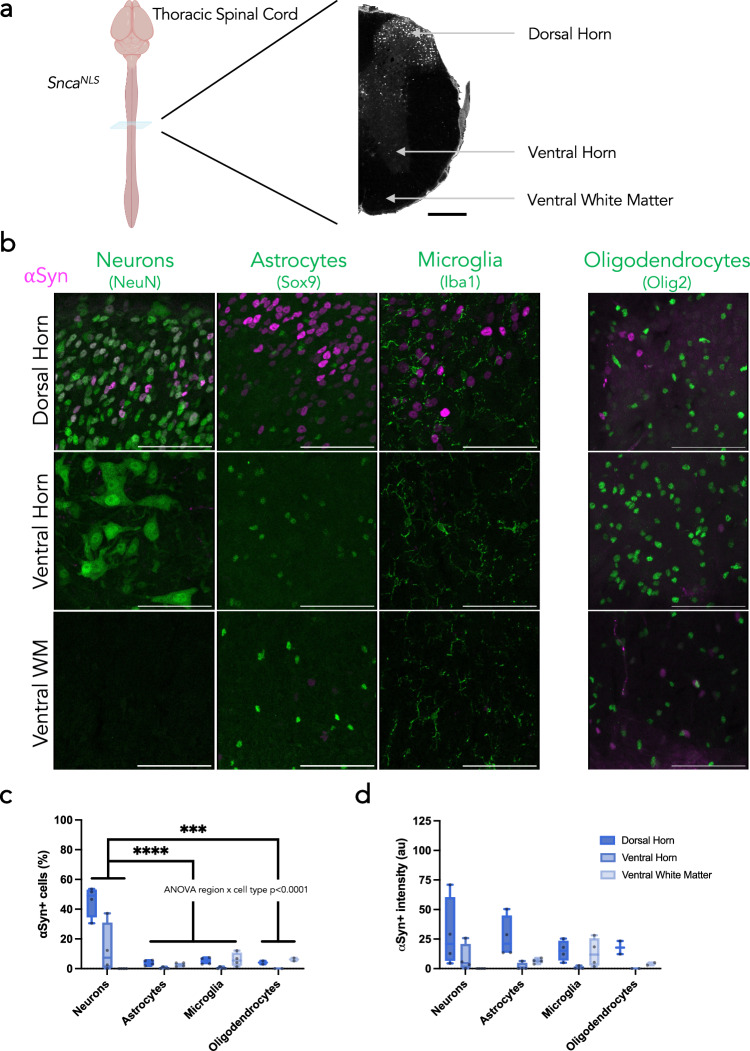
Diverse αSyn topography within the mouse spinal cord. **a** Coronal plane chosen to assess different regions of the thoracic spinal cord in *Snca*^*NLS*^ mice (left) with annotations for the dorsal and ventral horns and ventral white matter (right) from a section stained for αSyn. Scale bar: 1000 μm. **b** Merged micrographs from the dorsal horn (upper), ventral horn (middle), and ventral white matter (bottom) staining for neurons (left), astrocytes (middle left), microglia (middle right), and oligodendrocytes (right; different staining paradigm). Scale bars: 75 μm. From these, αSyn density (**c**) and intensity (**d**) were quantified. Plotted as box plot with min to max bars. Two-way ANOVA with Tukey’s post hoc, ***, **** denotes *p* < 0.001 and *p* < 0.0001, respectively.

The spinal cord is not routinely studied in PD, but has gained recognition for its position as a conduit between the gut and brain and its potential role in the gut-to-brain transmission of αSyn pathology^36,42–45^. Therefore, we next assessed αSyn staining patterns in the spinal cord of *Snca*^*NLS*^ mice. We analyzed sections from the cervical, thoracic and lumbar spinal cord (Fig. 4a, Supplementary Fig. 3a, 4a). Within these regions, we analyzed the dorsal horn, ventral horn, and ventral white matter. Similar to the brain, αSyn in the spinal cord is primarily neuronal; however, its localization is not homogenous (Fig. 4b, Supplementary Fig. 3b, 4b). αSyn is expressed primarily in the dorsal horn, more so in upper laminae, with αSyn+ cells only appearing in the ventral horn more caudally (Fig. 4c, Supplementary Fig. 3c). Additionally, αSyn intensity varies along the length of the cord, with the brightest, most concentrated αSyn+ cells existing in the thoracic region (Fig. 4d, Supplementary Fig. 3d). Nearly half of all NeuN+ cells of the thoracic and lumbar dorsal horn co-label with αSyn, a fraction significantly smaller than the neurons of the brain (>80%). Similar to the brain, however, astrocytes, microglia, and oligodendrocytes have very few cells that co-label with αSyn. Additionally, the intensity of αSyn in the dorsal horn is higher than that of the ventral horn and ventral white matter and is more consistent across cell types. These trends are all consistent in the cervical spinal cord (Supplementary Fig. 4).

To further investigate the neuronal localization of αSyn in the spinal cord, we co-stained cervical, thoracic, and lumbar sections with ChAT, a marker of motor neurons, and Pax2, a marker of GABAergic neurons. In both cases, we found that αSyn colocalizes with these markers primarily within lamina X surrounding the central canal (Supplementary Fig. 5). Since αSyn+ neurons represent only 50% of the entire spinal cord neuron population (NeuN+ ; compared to > 80% in the brain regions tested, Fig. 2), we turned to the harmonized atlas of single cell sequencing data to validate these findings^46,47^. Consistent with our imaging, we found that *Snca* was enriched in neurons (Supplementary Fig. 6a, a’, a”), specifically excitatory clusters 18 and 19 within the Sox5 family (distinguished by *Nmu* and *Tac2* expression, respectively), and inhibitory clusters 9–11 within the Pdyn family. Similarly, the harmonized atlas also illustrates the lack of overlap between *Snca* and motor neurons (Supplementary Fig. 6a”). Additionally, scRNA-Seq datasets from the mouse brain^48,49^ and colon^50,51^ show similar congruency with our atlas, illustrating the abundance of neuronal αSyn with specific sub-neuronal clusters exhibiting higher αSyn expression (Supplementary Fig. 6b–c”). These data demonstrate that αSyn exhibits a neuron subtype-specificity in the spinal cord.

### Olfactory bulb and retina show distinct subpopulations of cells with high αSyn density and intensity

**Fig. 5:**
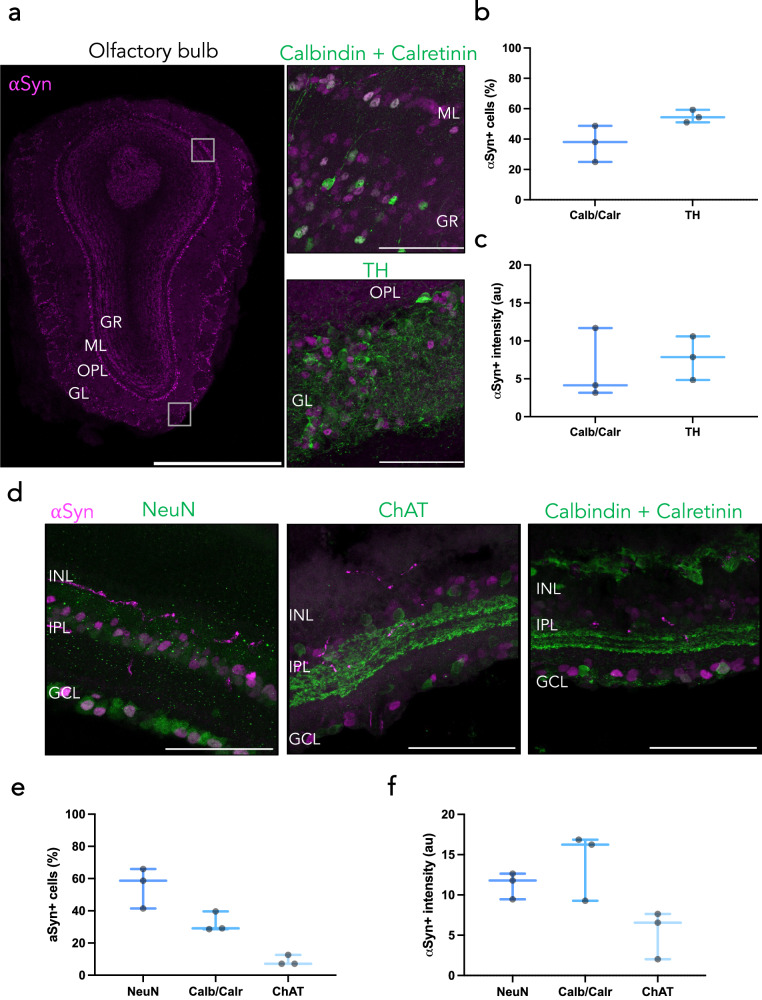
Diverse αSyn staining patterns within the mouse olfactory bulb and retina. **a** Tiled immunofluorescent image of an olfactory bulb (left; scale bar: 1000 μm) shows region-specific topography of αSyn, specifically colocalizing with calbindin/calretinin in the mitral layer (upper right) and TH in the glomerular layer (lower right). Scale bars: 75 μm. From these, αSyn density (**b**) and intensity (**c**) were quantified. Plotted as box plots with min to max bars. **d** Cross section of *Snca*^*NLS*^ retina co-stained for NeuN (left) ChAT (middle) or calbindin/calretinin (right). From these, αSyn density (**e**) and intensity (**f**) were quantified. Plotted as box plots with min to max bars. Glomerular layer (GL), outer plexiform layer (OPL), mitral layer (ML), granule layer (GR), ganglionic cell layer (GCL), inner plexiform layer (IPL), inner nuclear layer (INL).

The “brain-first” model of PD suggests that αSyn pathological spread begins in the brain, typically the olfactory bulb or amygdala, before moving into the periphery^52–54^. Additional in vivo work has implicated the olfactory bulb as a main hub for αSyn pathology, specifically in the mitral cells^55^. We therefore investigated the olfactory bulb in more detail in *Snca*^*NLS*^ mice. We found prominent αSyn staining in the mitral and glomerular regions of the olfactory bulb, where we found that αSyn colocalizes with about half of all TH+ glomerular cells and calbindin/calretinin+ cells in the granular and mitral layers (Fig. 5a–c).

In conjunction with the olfactory bulb, recent studies have explored the retina as a site for αSyn pathology genesis and spread in the brain-first model. Previous studies have shown increased αSyn pathology in the retina and optic nerve, with retinal neurodegeneration in PD and other α-synucleinopathy patients^56,57^. These studies typically report pathology and neurodegeneration in the ganglionic cell layer and inner plexiform layer. Incidentally, these sites of pathology are where we noted most αSyn+ cells. Upon co-staining with cell-specific markers, we noted αSyn does not colocalize with ChAT+ neurons, but with calbindin/calretinin+ neurons in the ganglion and inner plexiform layers (Fig. 5d–f). We also noted higher αSyn intensity in the ganglion cell layer than in the inner plexiform layer.

### All enteric neurons of the stomach and duodenum have αSyn protein of varying intensity

**Fig. 6:**
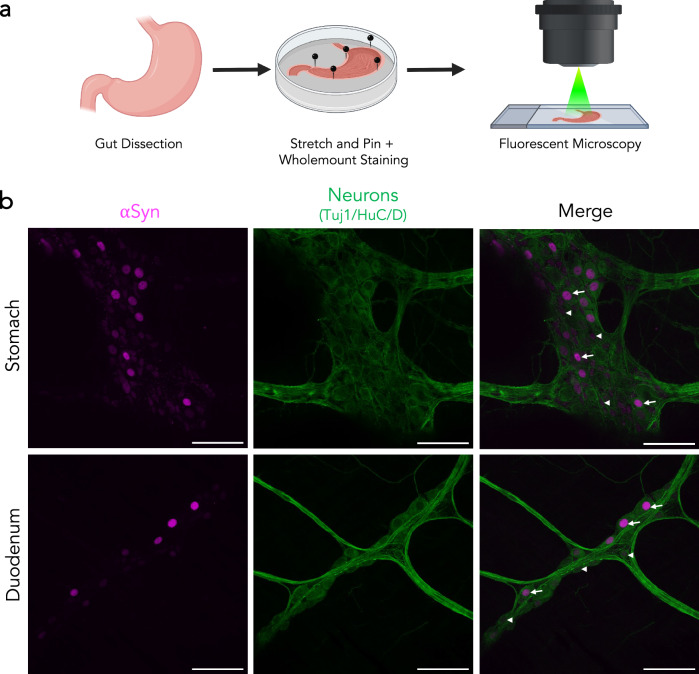
Varying intensities of αSyn expression throughout the gut. **a** *Snca*^*NLS*^ gut tissue was flushed and stained whole-mount prior to immunofluorescent staining and imaging. **b** αSyn shows 100% colocalization with neuronal markers in the stomach (upper) and duodenum (lower). Arrowheads denote low αSyn, arrows denote high αSyn intensity. Scale bars: 75 μm.

Gastrointestinal symptoms, including constipation, are known prodromal symptoms of PD and αSyn pathology has been observed in the gut tissue of PD patients^58–60^. We therefore aimed to characterize the cells that may be contributing to this pathological burden. Since we previously noted that αSyn primarily resides in neurons, we focused on the enteric neurons. We performed whole-mount staining of the stomach and duodenum of *Snca*^*NLS*^ mice then co-stained for αSyn and Tuj1/HuC/D to mark enteric neurons (Fig. 6a). This whole-mount preparation preserves both enteric neurons and longitudinal muscle. Here, we find αSyn exclusively within enteric neurons of both the stomach and duodenum with considerable variability in the relative amount of αSyn between neurons (Fig. 6b). This finding is consistent across the stomach and duodenum and is in line with recent enteric nervous system single-cell sequencing findings (Supplementary Fig. 6c, c’, c”)^51,61^.

## Discussion

In this study, we used our *Snca*^*NLS*^ mouse model to characterize the topography of αSyn systematically throughout the brain and in other PD-relevant tissues. We found that αSyn is expressed predominantly in neurons and that, while it is expressed in some of the non-neuronal cells tested, it is at much lower levels. To our surprise, cholinergic neurons express αSyn to varying degrees, with some expressing high levels of αSyn (e.g. DMX) while others are virtually devoid of αSyn (e.g. motor neurons of the spinal cord). It was interesting to note that olfactory neurons (particularly TH+ , periglomerular cells) were highly enriched for αSyn whereas retinal ganglion cells and amacrine cells expressed very low levels of αSyn, which bordered on the lower limit of detection. This is consistent with recent findings that suggest the olfactory bulb is likely a richer source of seed-competent αSyn than the retina however contrary to a 2016 study that found wildtype αSyn does not colocalize with TH+ cells in the periglomerular olfactory bulb^62–66^. This discrepancy may be due to the presynaptic/cytosolic localization of αSyn in wildtype mice whereby TH+ neurons in the periglomerular region of the olfactory bulb can project outside this layer, making interpretation of wildtype αSyn localization difficult. While our findings in mice are largely consistent with what is known of αSyn topography in the human brain, we acknowledge that these organisms may exhibit different patterns of αSyn localization. However, the patterns of αSyn expression we have identified may inform our understanding of human neuropathology. For example, the amygdala and substantia nigra pars compacta, which are heavily impacted by Lewy pathology in PD and other diseases, show high αSyn expression. In contrast, another region with high αSyn expression, the CA3 region of the hippocampus, is generally less impacted by Lewy pathology, suggesting that cellular resilience factors and neuronal connectivity also play a role in disease. However, when the hippocampus is impacted in patients, Lewy pathology is primarily in CA2/3, not CA1, consistent with higher expression of αSyn relating to higher likelihood of developing Lewy pathology^67–69^.

Astrocytes, microglia, and oligodendrocytes all expressed αSyn but at very low levels. Some populations of Olig2+ cells, however, expressed a considerable amount of αSyn. These are likely oligodendrocyte precursor cells (OPCs) which have previously been reported to express a relatively high level of αSyn^35,70–74^. The role that αSyn plays within these cells remains enigmatic, but higher native expression in OPCs, in concert with genetic and/or environmental insults, may predispose a cell to future pathology in diseases like MSA, where αSyn pathology principally occurs in oligodendrocytes.

Most αSyn+ cells of the spinal cord lie within the upper laminae of the dorsal horn, with very few αSyn+ cells in the ventral horn and ventral white matter. Concordantly, αSyn+ aggregates have been found in the dorsal horn of PD spinal cord tissue^75,76^. The upper laminae of the dorsal horn are largely comprised of the substantia gelatinosa (primarily GABAergic neurons) and the nucleus proprius (primarily somatostatin, calretinin, and VGLUT3+ neurons)^77^. These regions receive inputs from many higher order brain structures, including the locus coeruleus^78^. It has also been suggested that αSyn pathology spreads from the dorsal horn of the spinal cord to the dorsal root ganglia via the dorsal root^76^. However, additional work needs to be done to validate the specific cell types responsible for spreading, retaining αSyn pathology, and degenerating in disease.

Myenteric neurons of the enteric nervous system express a considerable amount of αSyn. Interestingly, this level is highly varied between neuronal subtypes. This finding is consistent with a recent single cell transcriptomic dataset that highlights an enrichment of *Snca* in enteric neuron classes (ENCs) 5, 9, 10 and 11^61^. Interestingly, ENC11 is defined by a high expression of TH and Calb2, markers that cluster with high αSyn expressing cells like dopamine neurons. This expression pattern could imply that it is these dopaminergic neurons of the gut that harbor more αSyn protein, and may be more vulnerable in disease, however additional research is required on the implications of αSyn protein load in the gut and how this translates to pathological burden in disease.

The development of this topographical atlas of αSyn expression sets the stage for future studies exploring the impact of neuronal αSyn on subsequent vulnerability to developing Lewy pathology. First, this atlas provides a newfound level of insight in healthy, young animals. Future work, looking at the impact of PD-relevant risk factors such as age, toxin or genetic exposure, or viral insults will shed important light on modes of native αSyn regulation. Second, it remains unclear whether the native abundance of αSyn affects the susceptibility of a cell to neurodegeneration. Seeding-based experiments suggest that cells with higher αSyn are more vulnerable to pathology^79,80^, while human imaging studies have suggested that regional atrophy in PD is related to anatomical connectivity^81^. Cellular milieu likely also plays a role in αSyn vulnerability, with studies finding different αSyn fibril strains forming in different cell types, leading to differing levels of pathogenicity^18^. It is likely that these factors (anatomical connectivity, gene expression, and cell environment) work in concert to make certain neurons and brain regions vulnerable to developing Lewy pathology^82^. Future work looking to selectively target αSyn-rich versus αSyn-poor circuits will provide much-needed understanding of the role of endogenous αSyn dosage and cellular vulnerability. Lastly, given the increased focus on αSyn-lowering therapeutics (e.g. immunotherapy, antisense oligonucleotides, aggregation inhibitors, viral approaches), this atlas will provide insight into why certain cell types may be more resistant to these translational modalities, providing ways to better target therapeutics and circumvent associated pitfalls.

## Methods

### Mouse husbandry

All mice were housed with up to 5 mice per cage on a 12 h light-dark cycle. Mice were fed *ad libitum* and all husbandry was performed by the uOttawa Animal Care and Veterinary Services staff. All animal work was performed under the breeding protocols (CMMb-3654 and CMMb-3904) approved by the uOttawa Animal Care Committee. The *Snca*^*NLS*^ mouse line used in this study is available through the Jackson Laboratory (036763; RRID:IMSR_Jax:036763). All housing and procedures at Van Andel Institute were performed according to the NIH Guide for the Care and Use of Experimental Animals and approved by the Van Andel Institute Institutional Animal Care and Use Committee (IACUC). C57BL/6 J mice were purchased from the Jackson Laboratory (000664; RRID:IMSR_JAX:000664). Both male and female mice were used in this study and all mice were between 9–13 weeks old.

### Tissue collection

Mice were sedated with an intraperitoneal injection of 120 mg/kg Euthanyl (DIN00141704). Next, they were transcardially perfused with 10 mL 1X phosphate buffered saline (PBS) + 10 U/mL heparin (Millipore Sigma, H3393–50KU) followed by 10 mL 4% paraformaldehyde (PFA). Brains were extracted and incubated in 4% PFA overnight at 4 ^o^C with gentle rocking. The brains were then washed twice with 1X PBS (dx.doi.org/10.17504/protocols.io.b5swq6fe). Brains used for Fig. 1a–c, and Supplementary Fig. 1 were processed into paraffin via sequential dehydration and perfusion with paraffin under vacuum (70% ethanol for 1 h, 80% ethanol for 1 h, 2 times 95% ethanol for 1 h, 3 times 100% ethanol for 1 h, 2 times xylene for 30 min, paraffin for 30 min at 60 °C, paraffin for 45 min at 60 °C). Brains were then embedded in paraffin blocks, cut into 6 µm sections and mounted on glass slides. Brains and spinal cords used for Figs. 2–5, and Supplementary Fig. 2–5 were dehydrated in serial sucrose solutions for 48 h each in 10%, 20%, then 30% sucrose. Next, the organs were flash frozen in ~ –40 ^o^C isopentane then cryosectioned at 20 μm and stored as free-floating tissue in 1X PBS with 0.02% sodium azide. Subsequent steps are protocol-dependent and outlined in downstream methods. See Supplementary Table 1 for a comprehensive list of all antibodies/kits used in this study.

### In situ hybridization

10.17504/protocols.io.bp2l61n91vqe/v1

In situ hybridization was performed with RNAscope® Multiplex Fluorescent Reagent Kit v2 (ACD, Cat #323270) using recommended conditions and supplied reagents. Paraffin-embedded tissue was freshly sectioned and dried. When it was not going to be used immediately, slides were vacuum-sealed and stored at 4 °C. Slides were baked in a dry oven for 1 h at 60 °C and used within one week. Slides were de-paraffinized with 2 sequential 5 min washes in xylenes, followed by 2 washes in 100% ethanol for 2 min. Slides were then dried for 5 min at 60 °C. Slides were treated with hydrogen peroxide for 10 min at room temperature and washed two times with distilled water. Target retrieval was performed in target retrieval reagents in the BioGenex EZ-Retriever System for 15 min at 99 °C. Slides were then washed with distilled water for 15 sec and transferred to 100% ethanol for 3 min before being dried for 5 min at 60 °C.

Slides were incubated in protease plus in a humidified tray in a hybridization oven (Boekel Scientific 240200) for 30 min at 40 °C. Slides were washed 2 times with distilled water. RNAscope® probes were added to slides and incubated for 2 h at 40 °C. The following probe was used: *Snca* (RNAscope probe, Mm-Snca-C1, 313281). Slides were washed twice for 2 min with wash buffer and incubated in Amp 1 for 30 min at 40 °C. The wash and Amp incubation was repeated for Amp 2 and Amp 3, except Amp 3 was only incubated for 15 min. Slides were washed twice for 2 min with wash buffer and incubated in HRP-C1 for 15 min at 40 °C. Slides were washed twice for 2 min with wash buffer and incubated in Opal 520 (Perkin Elmer FP1487A) for 30 min at 40 °C, washed twice for 2 min with wash buffer, incubated in HRP blocker for 15 min at 40 °C, and washed twice for 2 min with wash buffer. Slides were mounted with coverglass in ProLong gold with DAPI (Invitrogen, Cat#P36931) and imaged at 20X magnification on a Zeiss AxioScan 7 microscope.

### Immunofluorescence

10.17504/protocols.io.5jyl89m9dv2w/v1

Slides were de-paraffinized with 2 sequential 5 min washes in xylenes, followed by 1 min washes in a descending series of ethanols: 100%, 100%, 95%, 80%, 70%. Slides were then incubated in deionized water for one min prior and transferred to the BioGenex EZ-Retriever System where they were incubated in antigen unmasking solution (Vector Laboratories; Cat# H-3300) and microwaved for 15 min at 95 °C. Slides were allowed to cool for 20 min at room temperature and washed in running tap water for 10 min. Slides were washed for 5 min in 0.1 M Tris, then blocked in 0.1 M Tris/2% fetal bovine serum (FBS) for 2 h. Slides were incubated in primary antibody in 0.1 M Tris/2% FBS in a humidified chamber overnight at 4 °C.

Primary antibodies were rinsed off with 0.1 M tris and incubated in 0.1 M Tris/2% FBS for 5 min. Slides were then incubated in the dark for 3 h at room temperature with secondary antibodies in 0.1 M Tris/2% FBS. Slides were rinsed three times for 10 min in 0.1 M Tris in the dark, then mounted with coverglass in ProLong gold with DAPI (Invitrogen, Cat#P36931). Fluorescent slides were imaged at 20X magnification on a Zeiss AxioScan 7 microscope.

### Cell detection and classification

Our study included 99 sections from 4 *Snca*^*NLS*^ mice for immunofluorescence and 49 sections from 4 C57BL/6 J mice for in situ hybridization. Stained slides scanned on a Zeiss AxioScan 7 at 20x magnification were imported into QuPath (version 0.2.3, RRID:SCR_018257) or newer for analysis. Individual cells were identified using the Cell Detection feature that allows detection based on DAPI stain intensity. Cell detection parameters such as background radius, sigma, and threshold were adjusted to optimize cell detection across all brain sections.

After cell detection, cell classification was completed in QuPath using the object classification feature. QuPath was trained on a subset of annotations to distinguish between different cell types based on signal intensity. To train the classifier, cells were detected by DAPI then classified according to the nuclear expression intensity of αSyn staining or whole-cell intensity of *Snca* staining. Thresholds for the intensity classifications were calculated by averaging the mean intensity of all cells. Three classes were then generated: (1) Low: one standard deviation or less below the mean cellular intensity; (2) Medium: one standard deviation below or above the mean cellular intensity; (3) High: one standard deviation or more above the mean cellular intensity. This classifier was then applied to all brain sections. Each class was assigned a different color and exported as.png files for subsequent analysis.

### Mouse brain registration

Images were registered to the Allen Brain Atlas CCFv3 using a modified version of the QUINT workflow^21^. An RGB image of each section was exported from QuPath as a .png file, downsampled by a factor of 12, to use for spatial registration in QuickNII (RRID:SCR_016854)^22^. A segmentation image was created by exporting a color-coded image of classified cells by category on a white background to use as the segmentation input in Nutil (RRID:SCR_017183). Brain images were uploaded in Filebuilder and saved as an XML file to be compatible with QuickNII. Following the spatial registration of the mouse brain sections to the Allen Mouse Brain Atlas CCFv3 in QuickNII, a JSON file was saved for use in (VisuAlign, RRID:SCR_017978). Brain sections were imported into VisuAlign to perform non-linear warp transformations and align all brain regions. Anchor points were generated in the atlas overlay and moved to the corresponding location on the brain section. Final alignments were exported as .flat and .png files for use in Nutil^78^.

Nutil was used for the quantification and spatial analysis of the identified cell types in specific regions of the mouse brain. Individual classes were identified for quantification via their HTML color code assigned in QuPath. Nutil generated object counts from each individual classification within each region of the Allen Mouse Brain Atlas using the registration from QuickNII and VisuAlign. The percentage of each class was calculated for each region and used for subsequent plotting and analysis. Anatomical heatmaps were generated using custom R code (https://github.com/vari-bbc/Mouse_Brain_Heatmap; R project for statistical computing (RRID:SCR_001905) at https://www.r-project.org/). Raw output values and R code are available at https://zenodo.org/records/10685351.

#### DAB staining

10.17504/protocols.io.b5s9q6h6

Cryosectioned tissue were incubated in 0.9% hydrogen peroxide (Millipore Sigma, 216763–500 ML) for 10 min at room temperature. The following diaminobenzidine staining was performed using the Vectastain Elite ABC HRP Mouse Kit (Vector Laboratories, PK-6102). Briefly, tissue were incubated in blocking buffer (1.5% triton X-100 + 5% Vectastain Horse Serum in 1X PBS) for 1 h at room temperature following by incubation in primary antibody solution overnight at 4 ^o^C. Next, tissue was incubated in secondary antibody diluted in blocking buffer for 1 h, then ‘A + B’ solution (mixed 30 min prior to use) for 1 h at room temperature. Tissue was then exposed to diaminobenzidine for 2 min using the DAB Peroxidase HRP Substrate Kit (Vector Laboratories, SK-4100) then washed with 1X PBS. Tissue was dehydrated in 50%, 70%, 95%, then 100% ethanol for 1 min each, then 100% xylenes for 5 min. Finally, tissue was coverslipped with Permount mounting medium (Fisher Scientific, SP15–100) and #1.5 coverslips. Stained slides were imaged at 20X magnification using an Axio Scan Z1 Slide Scanner and exported as czi files for subsequent analysis.

### Hematoxylin staining

Cryosectioned tissue were stained using the H&E Staining Kit (Abcam, ab245880) following kit instructions. Briefly, free-floating sections were incubated in hematoxylin for 5 min then washed with 2 changes of water, blueing reagent for 15 sec, then washed in water again. Tissue was then mounted on a superfrost plus slide and dehydrated in 50%, 70%, 95%, then 100% ethanol for 1 min each then 100% xylenes for 5 min before coverslipping with Permount mounting medium (Fisher Scientific, SP15–100) and #1.5 coverslips. Stained slides were imaged at 20X magnification using the Axio Scan Z1 Slide Scanner and exported as .czi files for subsequent analysis.

### αSyn DAB Atlas quantification

All tissue slices were annotated to specific Allen Brain Atlas coordinates then converted to tiff files using Fiji ImageJ (version 2.3.0/1.53q, RRID:SCR_002285). The ‘pixel classification’ pipeline from ilastik software (version 1.3.2, RRID:SCR_015246) was used for to delineate positive cells and three images were used in training the machine learning software. Segmented images were opened in Fiji and brain regions were manually outlined and added to an ROI manager. The ‘analyze particles’ function was used to count positive cells greater than 5 pixels in size, corresponding to 1 positive cell. The number of αSyn+ cells was divided by the number of hematoxylin positive cells and multiplied by 100 to give then relative αSyn+ cell density.

### Whole brain clearing and imaging

Whole *Snca*^*NLS*^ mouse brains were processed using the SHIELD protocol by LifeCanvas Technologies^83^. Samples were cleared for 7 days with Clear+ delipidation buffer then immunolabeled using SmartLabel. Each sample was labeled with 5μg mouse anti-NeuN (RRID:AB_2572267) and 4μg goat anti-Flag (RRID:AB_299216) followed by fluorescent secondary antibodies. Samples were incubated in EasyIndex for a refractive index of 1.52 and imaged at 3.6X using SmartSPIM microscopy. Images were tile-corrected, de-striped, and registered to the Allen Brain Atlas (https://portal.brain-mapping.org; RRID:SCR_017001). The NeuN channel was registered to the 8–20 atlas-aligned reference samples using successive rigid, affine, and b-spline warping (SimpleElastix: https://simpleelastix.github.io). Average alignment to the atlas was generated across all intermediate reference sample alignments to serve as the final atlas alignment value per sample. Fluorescent measurements from the acquired images were projected onto the Allen Brain Atlas to quantify the total fluorescence intensity per region defined by the Allen Brain Atlas. These values were then divided by the volume of the corresponding regional volume to calculate the intensity per voxel measurements. Raw output files from LifeCanvas are available at https://zenodo.org/records/10685351. Data was plotted using Prism (version 9.5.1, RRID:SCR_002798) as a Box plot with min to max bars.

#### Organ isolation

10.17504/protocols.io.b5swq6fe

Following intracardial perfusion (see tissue collection method above), brains, spinal cords, and retina from *Snca*^*NLS*^ mice were extracted and incubated in 4% PFA for 72 h at 4 ^o^C with gentle rocking. Brain and spinal cords were next dehydrated in a 3-step sucrose sequence with 10, 20, and 30% sucrose for 24 h each. Finally, brains and spinal cord were flash frozen for 1 min at –40 ^o^C isopentane, suspended in OCT, then cryosectioned at 40 μm. Eyes were incubated in 70% ethanol following fixation, then retinas were isolated, suspended in OCT, and cryosectioned at 20 μm.

#### Immunofluorescence staining

10.17504/protocols.io.b5s5q6g6

Cryosectioned, free-floating tissue sections were incubated in blocking buffer (0.5% Triton X-100 (Sigma-Aldrich, T8787–100ML) + 10% normal horse serum (Sigma-Aldrich, H0146–5ML) in 1X PBS) for 1 h at room temperature. Next, sections were incubated in 1 ^o^ antibody overnight at 4 ^o^C then fluorescently conjugated 2 ^o^ antibody for 1 h at room temperature. Free-floating sections were mounted on SuperFrost + slides then coverslipped with fluorescent mounting medium (Agilent, S302380–2) and #1.5 coverslips.

#### ChAT staining

10.17504/protocols.io.rm7vzxbz4gx1/v1

Cryosectioned tissue were incubated in blocking buffer (0.1% Triton X-100 (Sigma-Aldrich, T8787–100ML) + 10% normal horse serum (Sigma-Aldrich, H0146–5ML) + 0.5% gelatin (Sigma-Aldrich, G1393–100ML) in 1X PBS) for 2 h at room temperature. Next, sections were incubated in 1 ^o^ antibody for 48 h at 4 ^o^C then fluorescently conjugated 2 ^o^ antibody for 2 h at room temperature. Free floating sections were mounted on SuperFrost + slides then coverslipped with fluorescent mounting medium (Agilent, S302380–2) and #1.5 coverslips.

### Brain quantification

Stained sections were imaged with a Zeiss LSM800 AxioObserver Z1 microscope at 20X with Z-stack. Images were then 3D projected and all channels were merged. Each αSyn + cell nucleus that co-labels with the specific cell marker was circled and the intensity was measured in Fiji ImageJ (version 2.3.0/1.53q) with the ROI manager. The background signal was measured by circling 3 separate regions of αSyn- space and measuring the intensity with the ROI manager. To quantify, the background values were averaged and subtracted from each cell-specific intensity measurement. These intensity values were plotted in a nested graph using Prism (version 9.5.1, RRID:SCR_002798) as a Box plot with min to max bars and analyzed using two-way ANOVA with Tukey’s post hoc analysis.

#### ENS immunofluorescence

10.17504/protocols.io.14egn3wxpl5d/v1

Following euthanasia and perfusion of animals, stomach and duodenum were collected, washed, stretched, pinned flat on Sylgard-coated Petri dishes, and fixed overnight at 4 °C in phosphate buffer saline solution (PBS) containing 4% (vol/vol) paraformaldehyde (cat# P6148, Sigma-Aldrich). Layers of tissue containing the myenteric plexus were separated by microdissection. Tissues were permeabilized at room temperature (RT) for 2 h in a 10% fetal bovine serum (FBS)/PBS blocking buffer containing 0.5% Triton X-100 (cat# X100, Sigma-Aldrich), and incubated overnight at 4 °C with the following primary antibodies diluted in the blocking buffer: rabbit monoclonal anti-α-synuclein. Following incubation with primary antibodies, tissues were washed with PBS and incubated for 1 h at RT in secondary antibodies. Tissues were then washed with PBS and mounted with ProLong Gold antifade reagent (cat# P36931, Thermo Fisher Scientific). Confocal images were acquired using a Nikon A1plus-RSi confocal microscopy under a 40X objective and were analyzed with Image J software (RRID:SCR_003070).

### Statistical analysis

All statistical analyses were performed using GraphPad Prism (version 9.5.1) unless otherwise specified using a Two-way ANOVA with Tukey’s post hoc or Unpaired t-test, specified in the methods above. All statistical analyses with all raw values that were used to generate each figure at https://zenodo.org/records/10685351.

### Reporting summary

Further information on research design is available in the Nature Research Reporting Summary linked to this article.

### Supplementary information

Reporting summary

Supplementary materials

## Data Availability

The datasets generated and/or analyzed during the current study are available on Zenodo at https://zenodo.org/records/10685351.

## Code availability

The underlying code for Fig. 1c is available with the raw data that went into that analysis at https://zenodo.org/records/10685351.
