# Supplementary material for: A topographical atlas of α-synuclein dosage and cell type-specific expression in adult mouse brain and peripheral organs
Source: NPJ Parkinsons Dis. 2024 Mar 19;10:65. doi: 10.1038/s41531-024-00672-8 (PMC10951202; doi:10.1038/s41531-024-00672-8)
Supplement: Supplementary file 2 — Supplementary materials [file 41531_2024_672_MOESM2_ESM.pdf]

Supplementary materials for

A topographical atlas of α-synuclein dosage and cell type-specific expression in adult mouse brain and peripheral organs

Haley M. Geertsma[1,2,3,*], Zoe A. Fisk[1,2,3,*], Lillian Sauline[3,4,*], Alice Prigent[3,4], Kevin Kurgat[3,4], Steve M. Callaghan[1,2,3], aSCENT-PD Consortium[3], Michael X. Henderson[3,4,#], Maxime W.C. Rousseaux[1,2,3,#]

[1] University of Ottawa Brain and Mind Research Institute, Ottawa, ON, K1H8M5

[2] Department of Cellular and Molecular Medicine, University of Ottawa, Ottawa, ON, K1H8M5

[3] Aligning Science Across Parkinson's (ASAP) Collaborative Research Network, Chevy Chase, MD, USA

[4] Department of Neurodegenerative Science, Van Andel Institute, Grand Rapids, MI, USA

[*] Authors contributed equally
[#] Co-corresponding authors
Correspondence: max.rousseaux@uottawa.ca or michael.henderson@vai.org

aSCENT-PD Consortium: Benjamin R. Arenkiel[3,6], Brit Mollenhauer[3,7], Michael G. Schlossmacher[3,8], Christine Stadelmann[3,9], Julianna J. Tomlinson[3,8]

[6] Department of Molecular and Human Genetics, Baylor College of Medicine, Houston, TX 77030

[7] Paracelsus-Elena-Klinik, Kassel, Germany

[8] Program in Neuroscience, Ottawa Hospital Research Institute, University of Ottawa Brain and Mind Research Institute, Ottawa, ON, Canada K1H8M5

[9] Institute of Neuropathology, University Medical Center Göttingen, Göttingen, Germany

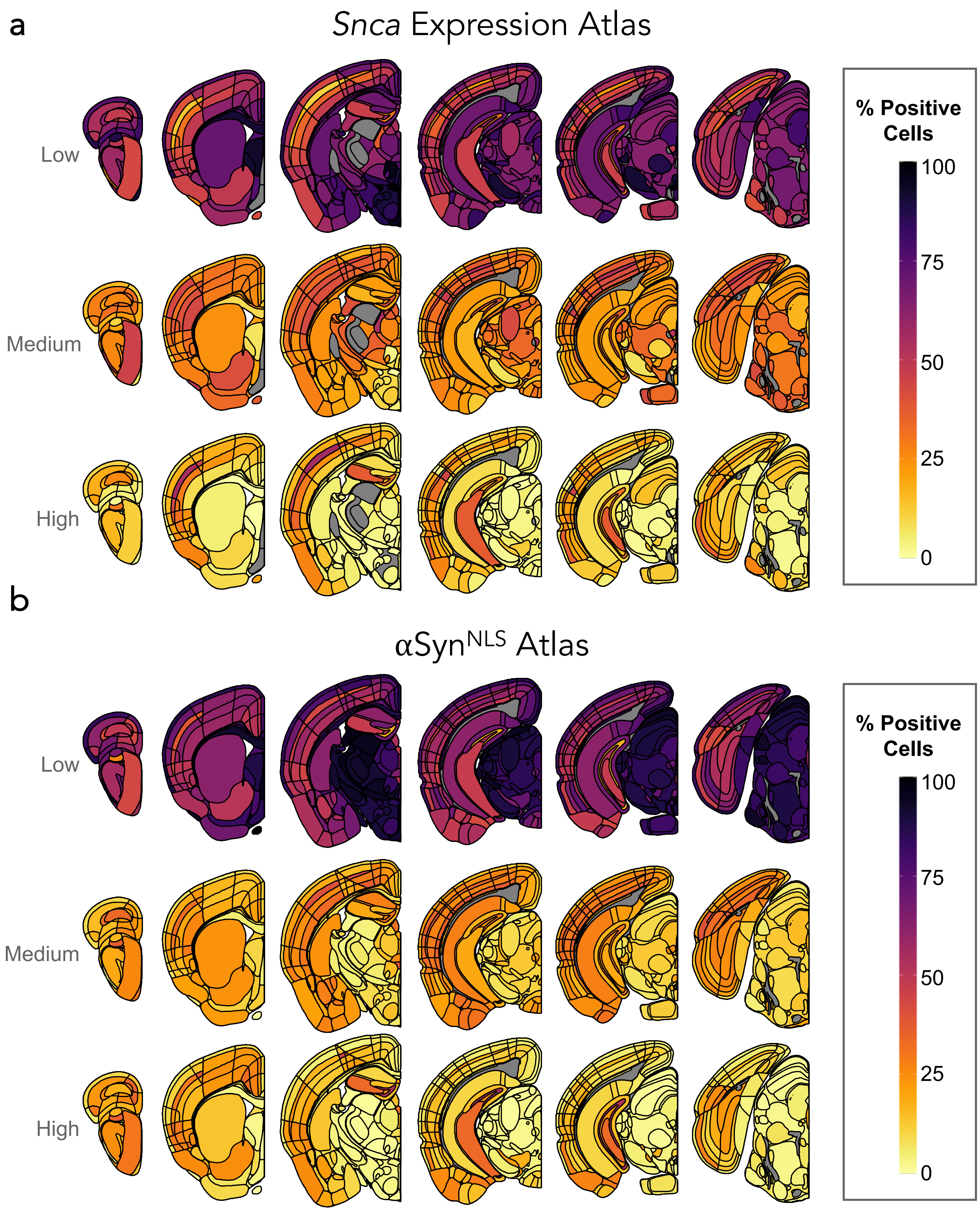

a
Snca Expression Atlas
Low
Medium
High
% Positive Cells
100
75
50
25
0
b
αSyn^{NLS} Atlas
Low
Medium
High
% Positive Cells
100
75
50
25
0

Supplementary Figure 1: Brain-wide heatmaps of *Snca* expression and αSyn protein density. a) *Snca* intensity from RNAScope of a wild-type mouse binned into low (upper panel), medium (middle panel), and high intensity (lower panel) prior to generating the heatmaps. **b**) αSyn staining intensity of a $\textit{Snca}^{\textit{NLS}}$ mouse binned into low (upper panel), medium (middle panel), and high intensity (lower panel) prior to generating the heatmaps.

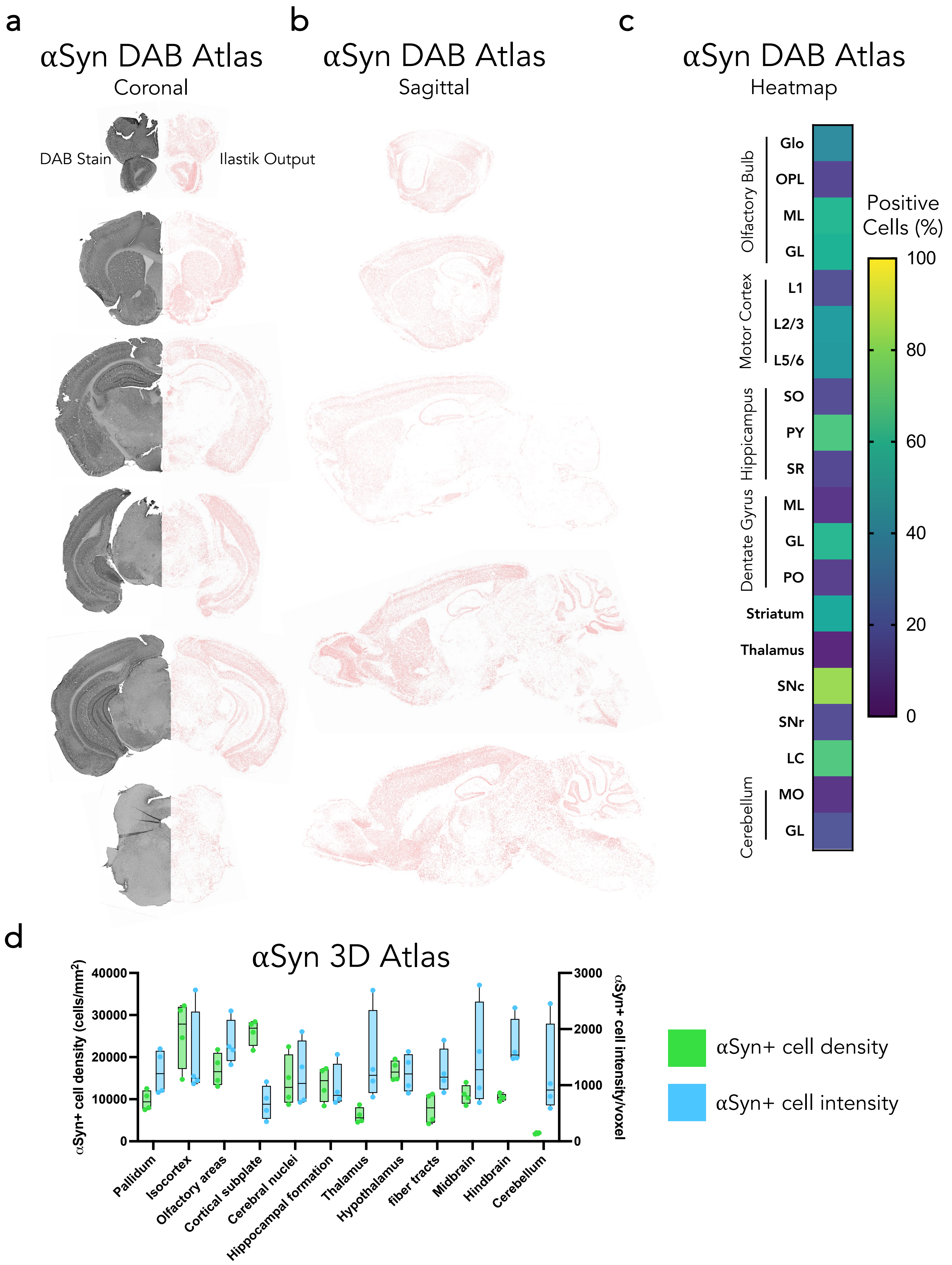
a
αSyn DAB Atlas
Coronal
DAB Stain
Ilastik Output
b
αSyn DAB Atlas
Sagittal
c
αSyn DAB Atlas
Heatmap
Olfactory Bulb
Glo
OPL
ML
GL
Motor Cortex
L1
L2/3
L5/6
Hippicampus
SO
PY
SR
Dentate Gyrus
ML
GL
PO
Striatum
Thalamus
SNc
SNr
LC
Cerebellum
MO
GL
Positive Cells (%)
100
80
60
40
20
0
d
αSyn 3D Atlas
αSyn+ cell density (cells/mm²)
40000
30000
20000
10000
0
αSyn+ cell intensity/voxel
3000
2000
1000
0
Pallidum
Isocortex
Olfactory areas
Cortical subplate
Cerebral nuclei
Hippocampal formation
Thalamus
Hypothalamus
fiber tracts
Midbrain
Hindbrain
Cerebellum
αSyn+ cell density
αSyn+ cell intensity

Supplementary Figure 2: Brain-wide atlas of αSyn protein density and intensity. a) Coronal atlas of αSyn DAB staining (left) and ilastik segmentation (right). **b**) Sagittal atlas of αSyn ilastik segmentation following DAB staining. **c**) Quantification of αSyn density in targeted brain regions. **d**) Quantification of αSyn+ density and intensity from whole brain staining and imaging. Plotted as box plots with min to max bars.

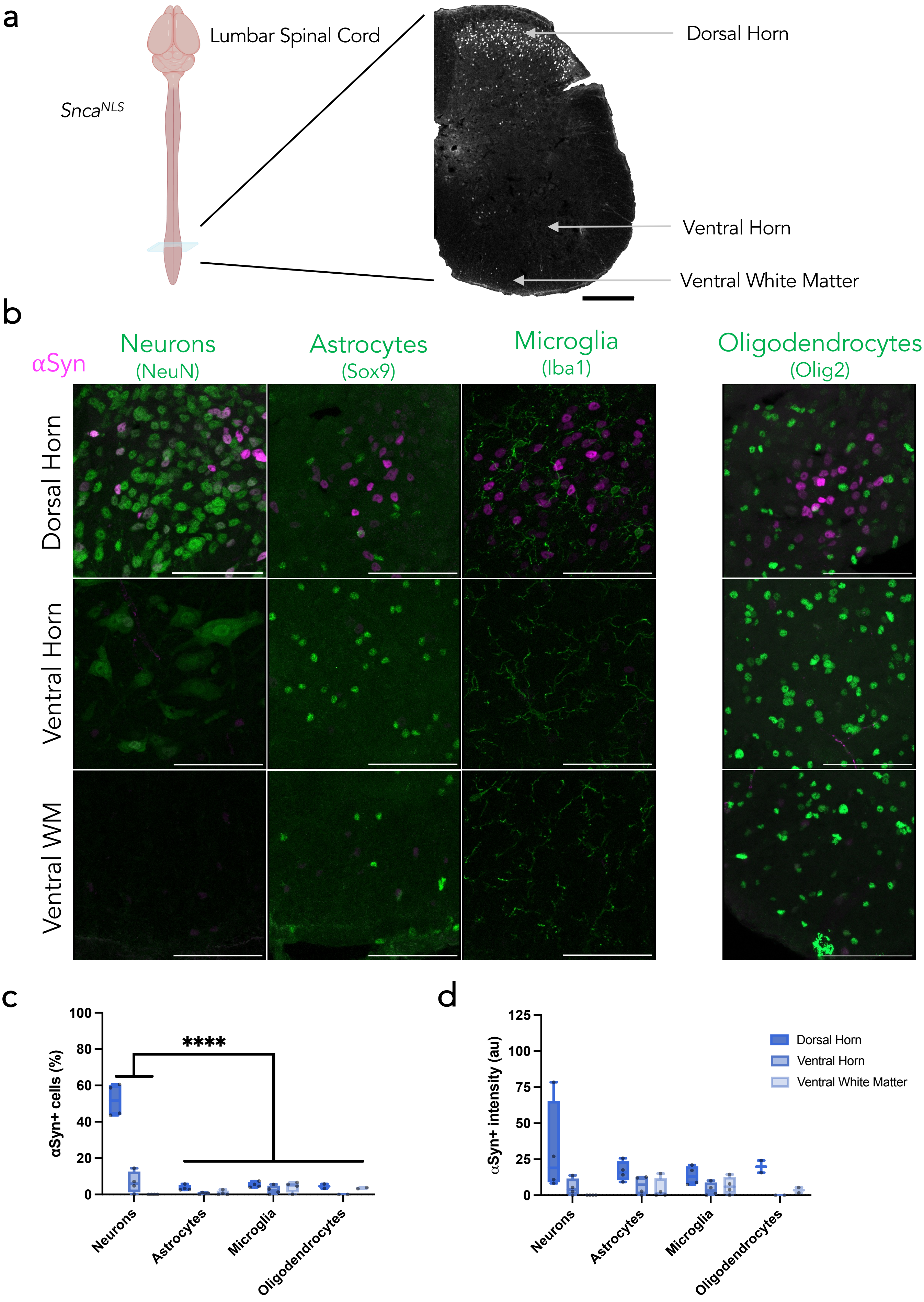
a
Lumbar Spinal Cord
SncaNLS
Dorsal Horn
Ventral Horn
Ventral White Matter
b
αSyn
Neurons
(NeuN)
Astrocytes
(Sox9)
Microglia
(Iba1)
Oligodendrocytes
(Olig2)
Dorsal Horn
Ventral Horn
Ventral WM
c
αSyn+ cells (%)
100
80
60
40
20
0
****
Neurons
Astrocytes
Microglia
Oligodendrocytes
d
αSyn+ intensity (au)
125
100
75
50
25
0
Dorsal Horn
Ventral Horn
Ventral White Matter
Neurons
Astrocytes
Microglia
Oligodendrocytes

Supplementary Figure 3: αSyn density and intensity of the lumbar spinal cord. a) Coronal plane chosen to assess different regions of the lumbar spinal cord in *Snca*^{NLS} mice (left) with annotations for the dorsal and ventral horns and ventral white matter (right) from a section stained for αSyn. Scale bar: 1,000µm. **b**) Merged micrographs from the dorsal horn (upper), ventral horn (middle), and ventral white matter (bottom) staining for neurons (left), astrocytes (middle left), microglia (middle right), and oligodendrocytes (right; different staining paradigm). From these, αSyn density (**c**) and intensity (**d**) were quantified. Scale bars: 75µm. Plotted as box plot with min to max bars. Two-way ANOVA with Tukey's post hoc, **** denotes $p<0.0001$.

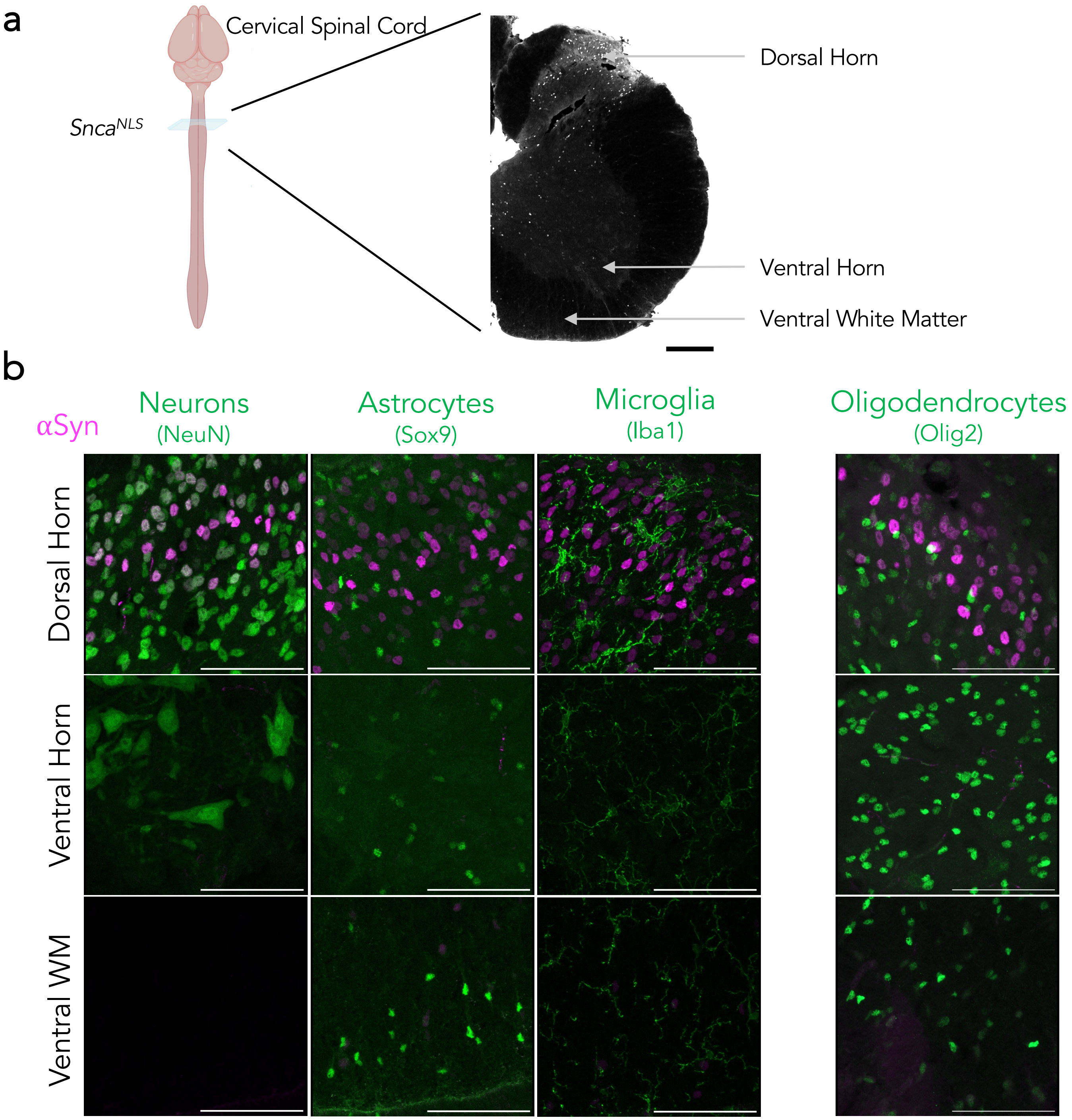

a
Cervical Spinal Cord
Snca^NLS
Dorsal Horn
Ventral Horn
Ventral White Matter
b
αSyn
Neurons
(NeuN)
Astrocytes
(Sox9)
Microglia
(Iba1)
Oligodendrocytes
(Olig2)
Dorsal Horn
Ventral Horn
Ventral WM

Supplementary Figure 4: αSyn staining of the cervical spinal cord. a) Coronal plane chosen to assess different regions of the lumbar spinal cord in *Snca*^{NLS} mice (left) with annotations for the dorsal and ventral horns and ventral white matter (right) from a section stained for αSyn. Scale bar: 1,000μm. **b**) Merged micrographs from the dorsal horn (upper), ventral horn (middle), and ventral white matter (bottom) staining for neurons (left), astrocytes (middle left), microglia (middle right), and oligodendrocytes (right; different staining paradigm). Scale bars: 75μm.

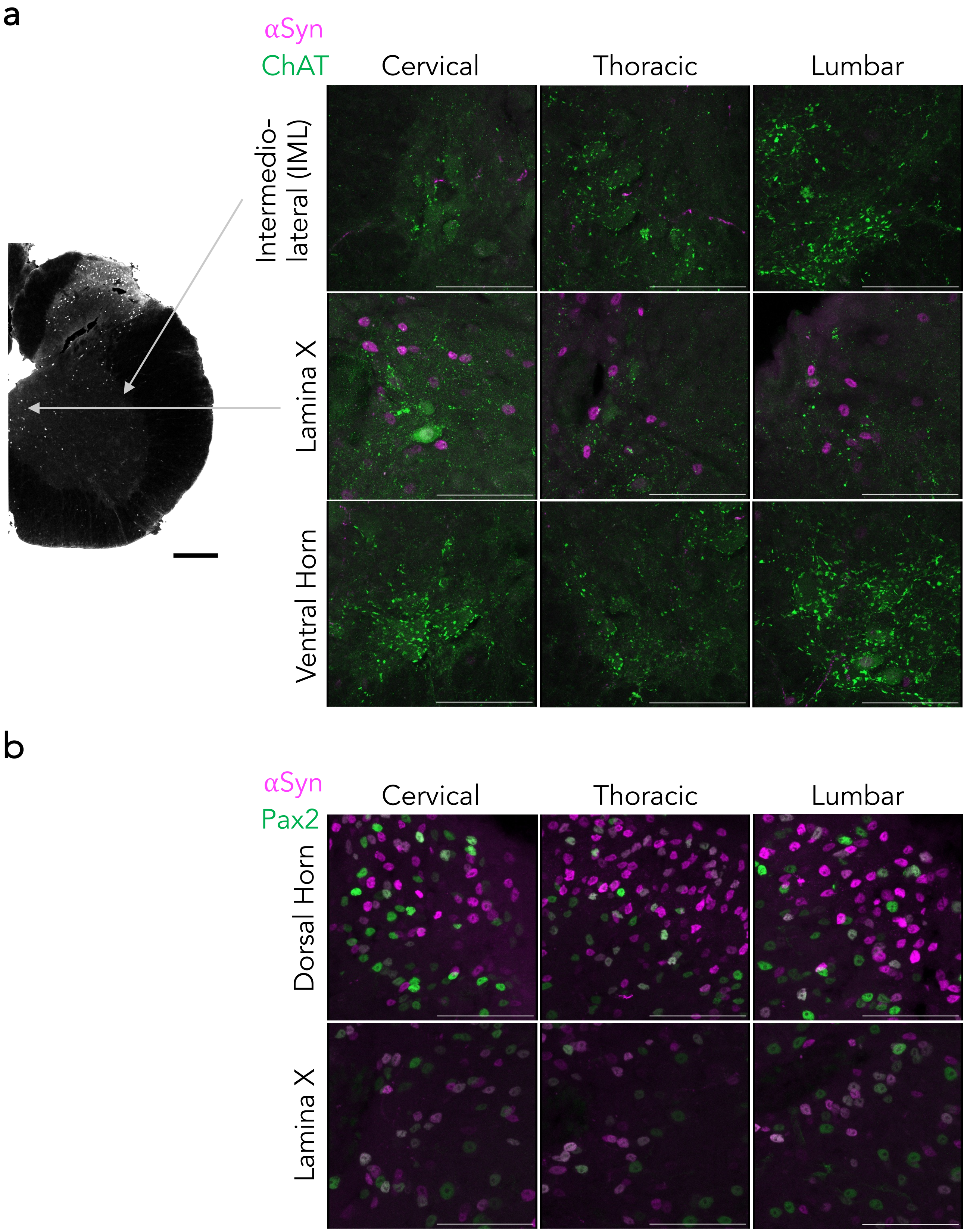
a
αSyn
ChAT
Cervical
Thoracic
Lumbar
Intermedio-lateral (IML)
Lamina X
Ventral Horn
b
αSyn
Pax2
Cervical
Thoracic
Lumbar
Dorsal Horn
Lamina X

Supplementary Figure 5: αSyn staining patterns throughout the spinal cord with neuron-specific markers for ChAT and Pax2. a) Coronal plane chosen to assess cervical (left) thoracic (middle) or lumbar (right) *Snca*^{NLS} spinal cord in the intermediolateral (upper), lamina X (middle), and ventral horn (lower) co-stained for αSyn and ChAT. Scale bar or coronal plane: 1000μm. Scale bars for immunofluorescent images: 75μm. **b**) Coronal plane chosen to assess cervical (left) thoracic (middle) or lumbar (right) *Snca*^{NLS} spinal cord in the dorsal horn (upper) and ventral horn (lower) co-stained for αSyn and Pax2. Scale bars: 75 μm.

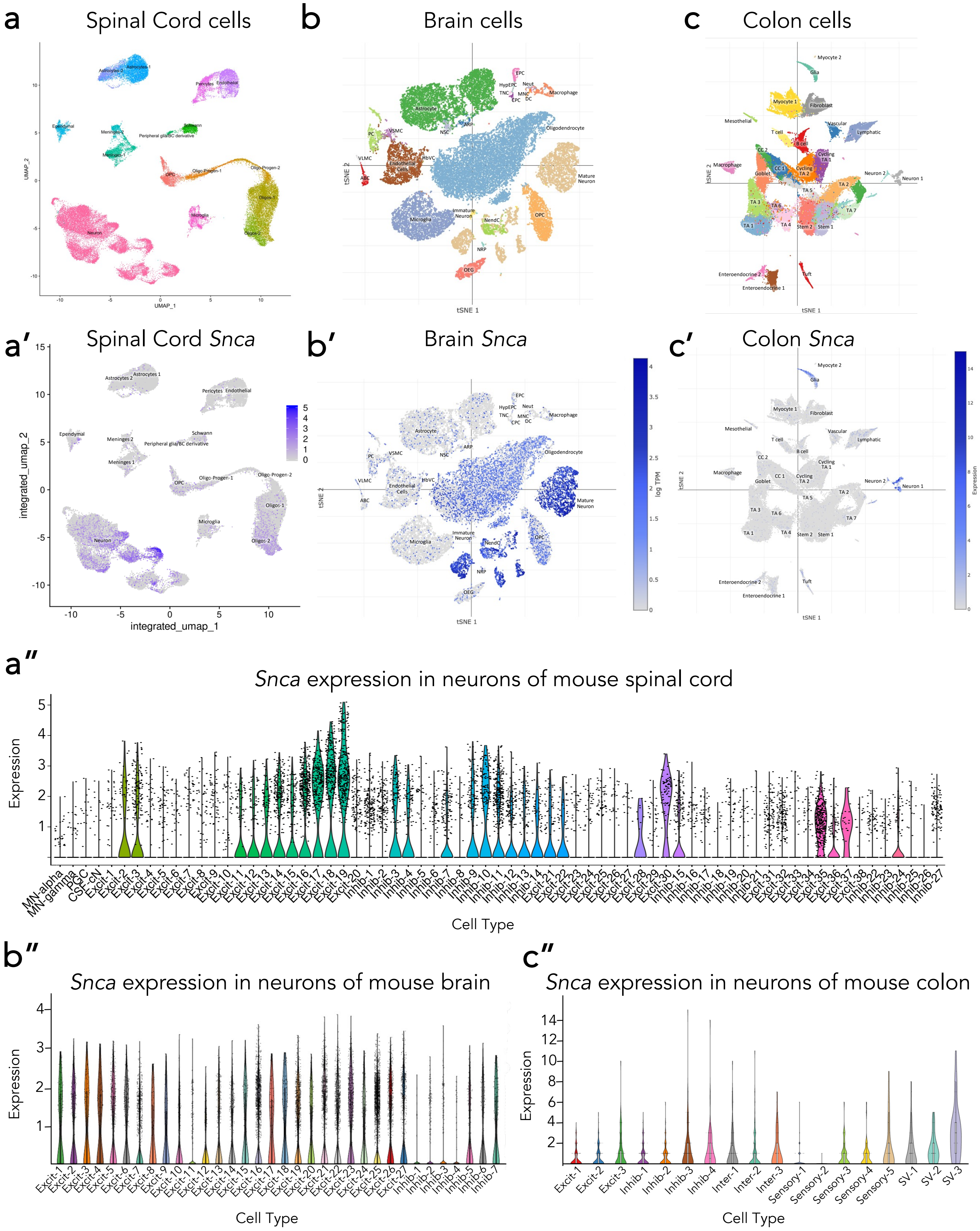

a
Spinal Cord cells
b
Brain cells
c
Colon cells
a′
Spinal Cord *Snca*
b′
Brain *Snca*
c′
Colon *Snca*
a″
Snca expression in neurons of mouse spinal cord
Expression
Cell Type
MN-alpha
MN-gamma
PGC
CSF-cN
Excit-1
Excit-2
Excit-3
Excit-4
Excit-5
Excit-6
Excit-7
Excit-8
Excit-9
Excit-10
Excit-11
Excit-12
Excit-13
Excit-14
Excit-15
Excit-16
Excit-17
Excit-18
Excit-19
Excit-20
Inhib-1
Inhib-2
Inhib-3
Inhib-4
Inhib-5
Inhib-6
Inhib-7
Inhib-8
Inhib-9
Inhib-10
Inhib-11
Inhib-12
Inhib-13
Inhib-14
Excit-21
Excit-22
Excit-23
Excit-24
Excit-25
Excit-26
Excit-27
Excit-28
Excit-29
Excit-30
Inhib-15
Inhib-16
Inhib-17
Inhib-18
Inhib-19
Inhib-20
Inhib-21
Excit-31
Excit-32
Excit-33
Excit-34
Excit-35
Excit-36
Excit-37
Excit-38
Inhib-22
Inhib-23
Inhib-24
Inhib-25
Inhib-26
Inhib-27
b″
Snca expression in neurons of mouse brain
Expression
Cell Type
c″
Snca expression in neurons of mouse colon
Expression
Cell Type
Excit-1
Excit-2
Excit-3
Inhib-1
Inhib-2
Inhib-3
Inhib-4
Inter-1
Inter-2
Inter-3
Sensory-1
Sensory-2
Sensory-3
Sensory-4
Sensory-5
SV-1
SV-2
SV-3

Supplementary Figure 6: Mining scRNA-Seq datasets to explore αSyn expression patterns in the mouse spinal cord, brain, and colon. **a**) UMAP plot from the Harmonized Atlas of Mouse Spinal Cord with **a'**) *Snca* expression pattern and **a"**) neuron-specific sub-clustering. **b**) tSNE plot from the Aging Mouse Brain dataset from the Broad Institute's Single Cell Portal with **b'**) *Snca* expression pattern and **b"**) neuron-specific sub-clustering from the study Dissecting Cell-Type Composition and Activity-Dependent Transcriptional State in Mammalian Brains by Massively Parallel Single-Nucleus RNA-Seq. **c**) tSNE plot from the Human and Mouse Enteric Nervous System at Single Cell Resolution from the Broad Institute's Single Cell Portal with **c'**) *Snca* expression pattern and **c"**) neuron-specific sub-clustering. ABC (arachnoid barrier cells), ARP (astrocyte-restricted precursors), CC (colonocyte), CPC (choroid plexus epithelial cells), CSF-cN (cerebrospinal fluid contacting neurons), DC (dendritic cells), EPC (ependymocytes), Excit- (excitatory neurons), HbVC (hemoglobin-expressing vascular cells), HypEPC (hypependymal cells), Inhib- (inhibitory neurons), Inter- (interneurons), MN (motor neurons), MNC (monocytes), NendC (neuroendocrine cells), Neut (neutrophils), NRP (neuronal-restricted precursors), NSC (neural stem cells), OEG (olfactory ensheathing glia), OPC (oligodendrocyte precursor cells), PC (pericytes), PGC (preganglionic cells), Sensory (sensory neurons), Stem (stem cells), SV (secretomotor/vasodilator neurons), TA (transit-amplifying cells), TNC (tanycytes), VLMC (vascular and leptomeningeal cells).

Supplementary Table 1 Antibodies used in this study with their application

	Antibodies (alphabetical)	Provider, Catalog #, RRID	Species	Application	Concentration
**Primary**	αSynuclein	BD Biosciences, 610787, RRID:AB_398108	Mouse	Immunofluoresence, αSyn^{NLS} Atlas	1:2000
				Immunofluoresence, cell-specific staining & Immunohistochemistry, αSyn DAB Atlas	1:1000
		Cell Signaling Technology, 4179, RRID:AB_1904156	Rabbit	ENS immunofluoresence	1:250
	Calbindin D28K	Invitrogen, PA5-85669, RRID:AB_2792808	Rabbit	Immunofluoresence, cell-specific staining	1:1000
	Calretinin	Invitrogen, PA5-143562, RRID:AB_2942791	Chicken	Immunofluoresence, cell-specific staining	1:1000
	Choline acetyltransferase (ChAT)	EMD Millilpore, AB144P, RRID:AB_2079751	Goat	Immunofluoresence, cell-specific staining	1:200
	DAPI	Millipore Sigma, D9542	-	Immunofluoresence, cell-specific staining	1:1000
	Flag (DDDDK)*	Abcam, ab1257, RRID:AB_299216	Goat	Immunofluoresence, αSyn 3D Atlas	4µg
	HuC/HuD	ThermoFisher Scientific, A-21271, RRID:AB_221448	Mouse	ENS immunofluoresence	1:100
	Iba1	Fujifilm Wako, 019-19741, RRID:AB_839504	Rabbit	Immunofluoresence, cell-specific staining	1:1000
	NeuN	Millipore Sigma, 3881957, RRID:AB_10807954	Rabbit	Immunofluoresence, αSyn^{NLS} Atlas	1:500
		Cell Signaling Technologies, 12943S, RRID:AB_2630395	Rabbit	Immunofluoresence, cell-specific staining	1:1000
		EnCor Biotechnology, MCA-1B7, RRID:AB_2572267	Mouse	Immunofluoresence, αSyn 3D Atlas	5µg
	Olig2	R&D Systems, AF2418, RRID:AB_2157554	Goat	Immunofluoresence, cell-specific staining	1:200
	Pax2	R&D Systems, AF3364-SP, RRID:AB_10889828	Goat	Immunofluoresence, cell-specific staining	1:200
	Sox9	Abcam, ab185966, RRID:AB_2728660	Rabbit	Immunofluoresence, cell-specific staining	1:1000
	Tuj1 (βIII-tubulin)	Sigma-Aldrich, T8660, RRID:AB_477590	Mouse	ENS immunofluoresence	1:500
	Tyrosine hydroxylase	Millipore, AB152, RRID:AB_390204	Rabbit	Immunofluoresence, cell-specific staining	1:1000
**Secondary**	Donkey-anti-Goat Alexa Fluor 488	ThermoFisher Scientific, A32814, RRID:AB_2762838	Goat	Immunofluoresence	1:500
	Goat-anti-Mouse IgG (H+L) Alexa Fluor 488	Thermo Fisher Scientific, A-11001, RRID:AB_2534069	Mouse		
	Goat-anti-Mouse IgG2b Alexa Fluor 568	Thermo Fisher Scientific, A-21144, RRID:AB_2535780			
	Donkey-anti-Mouse Alexa Fluor 594	ThermoFisher Scientific, R37115, RRID:AB_2556543			
	Goat-anti-Rabbit IgG (H+L) Alexa Fluor 647	ThermoFisher Scientific, A-21244, RRID:AB_2535812	Rabbit		
	Goat-anti-Chicken IgY (H+L) Alexa Fluor 647	Thermo Fisher Scientific, A-21449, RRID:AB_2535866	Chicken		
	Goat-anti-Mouse IgG1 Alexa Fluor 488	Invitrogen, A21121, RRID:AB_2535764	Mouse		1:1000
	Goat-anti-Rabbit Alexa Fluor 546	Invitrogen, A11010, RRID:AB_2534077	Rabbit		
**Staining Kits**	VectaStain Elite ABC Peroxidase Kit	Vector Laboratories, PK-6102, RRID:AB_2336821	Mouse	Immunohistochemistry, αSyn DAB Atlas	-
	DAB Substrate Kit, Peroxidase HRP, with Nickel (3,3'-diaminobenzidine)	Vector Laboratories, SK-4100, RRID:AB_2336382	-	Immunohistochemistry, αSyn DAB Atlas	-

*this antibody has since been discontinued
